# The causes of bullying: results from the National Survey of School Health (PeNSE)

**DOI:** 10.1590/0104-1169.0022.2552

**Published:** 2015-04-14

**Authors:** Wanderlei Abadio de Oliveira, Marta Angélica Iossi Silva, Flávia Carvalho Malta de Mello, Denise Lopes Porto, Andréa Cristina Mariano Yoshinaga, Deborah Carvalho Malta

**Affiliations:** 1 Doctoral student, Escola de Enfermagem de Ribeirão Preto, Universidade de São Paulo, WHO Collaborating Centre for Nursing Research Development, Ribeirão Preto, SP, Brazil; 2 PhD, Associate Professor, Escola de Enfermagem de Ribeirão Preto, Universidade de São Paulo, WHO Collaborating Centre for Nursing Research Development, Ribeirão Preto, SP, Brazil; 3 MSc, Statistician, Coordenação Geral de Informações e Análise Epidemiológica, Secretaria de Vigilância em Saúde, Ministério da Saúde, Brasília, DF, Brazil; 4 Master´s student, Escola de Enfermagem de Ribeirão Preto, Universidade de São Paulo, WHO Collaborating Centre for Nursing Research Development, Ribeirão Preto, SP, Brazil; 5 PhD, Adjunct Professor, Escola de Enfermagem, Universidade Federal de Minas Gerais, Belo Horizonte, MG, Brazil. Director, Departamento de Vigilância de Doenças e Agravos não Transmissíveis e Promoção da Saúde, Secretaria de Vigilância em Saúde, Ministério da Saúde, Brazil

**Keywords:** School Health, Adolescent Health, Causality, Violence, Bullying

## Abstract

**Objective:**

to identify the characteristics and reasons reported by Brazilian students for school bullying.

**Method:**

this cross-sectional study uses data from an epidemiological survey (National Survey of School Health) conducted in 2012. A total of 109,104 9th grade students from private and public schools participated. Data were collected through a self-applied questionnaire and the analysis was performed using SPSS, version 20, Complex Samples Module.

**Results:**

the prevalence of bullying was 7.2%, most frequently affecting Afro-descendant or indigenous younger boys, whose mothers were characterized by low levels of education. In regard to the reasons/causes of bullying, 51.2% did not specify; the second highest frequency of victimization was related to body appearance (18.6%); followed by facial appearance (16.2%); race/color (6.8%); sexual orientation 2.9%; religion 2.5%; and region of origin 1.7%. The results are similar to those found in other sociocultural contexts.

**Conclusion:**

the problem belongs to the health field because it gathers aspects that determine the students' health-disease-care continuum.

## Introduction

The term bullying refers to a specific form of aggressive and violent behavior among peers in the school context. It is characterized by three criteria: intentionality, repeatability and imbalance of power^(^ 1 ^)^. Given the emphasis of this definition, school bullying are acts that repeat over time and involve a desire to harm colleagues or expose them to negative situations, while those exposed to negative situations have difficulty defending themselves^(^ 1 ^-^ 2 ^)^. This phenomenon may manifest directly and physically (e.g., hitting, spitting), verbally (derogatory nicknames, threats, insults, gossip), or through cyber-bullying (using social, electronic or communication media - internet, phone) or indirectly in situations where there is no direct confrontation among those involved (social exclusion, gossip)^(^ 3 ^-^ 4 ^)^.

Bullying is acknowledged as a relationship problem in which power is claimed through the use of violence and is a reality among school-aged children and adolescents in different cultural contexts^(^ 4 ^)^ and a severe problem in many countries^(^ 3 ^-^ 5 ^)^. This phenomenon may lead students to experience psychological distress, compromise the teaching-learning process and influence how individuals respond to social demands over the course of their lives. These negative consequences^(^ 4 ^,^ 6 ^)^, entailed for all those involved and associated with increased prevalence and frequency with which bullying occurs^(^ 7 ^-^ 8 ^)^, transformed bullying into a severe public health problem worldwide^(^ 9 ^-^ 10 ^)^.

Studies show that both boys and girls become involved in situations of violence at school, though the actions in which they engage are different. Boys are more likely to experience physical bullying, while girls engage in indirect or verbal exchanges^(^ 1 ^,^ 5 ^,^ 10 ^)^. Even though there are an increased number of studies addressing school bullying, few of them address causal factors or the reasons determining the phenomenon. In general, the focus of investigations is on the characteristics of the students involved, the phenomenon's variables and the nuances it assumes in the school context without, however, establishing the reasons that explain this phenomenon.

In this sense, evidence from the scientific literature addressing this subject suggests that the dynamics of bullying is a result of the students' characteristics, the vulnerability or social status of one student in relation to another, that differentiate and segregate peers^(^ 3 ^)^. A study conducted in Netherlands with 80,770 students reports that the reasons students presented for the practice of bullying were physical appearance, individual behavior, level of school performance, physical or mental disabilities, religious aspects, gender issues, sexual orientation, and the inappropriate manner some students dealt with punishment^(^ 11 ^)^. The average prevalence of students identified as involved in bullying was 32.5%^(^ 11 ^)^.

A longitudinal studied conducted in the United States reports empirical evidence of increased school bullying beginning in the second half of the 2000s, with a prevalence of 25.8% in 2009^(^ 12 ^)^. The study reports bullying was more common and more intense among boys, Afro-descendants, from rural areas, living with single parents, with low school performance and a low level of religious identification^(^ 12 ^)^. A Swedish study, reporting a prevalence of 44% of victims and aggressors, reports that adolescents tend to explain the phenomenon in terms of individual reasons instead of offering other dimensions like peer groups, school context, or social issues^(^ 5 ^)^. The study also reveals that aggressors were more likely to blame the victim^(^ 5 ^)^.

In Brazil, the complexity of concrete problems such as bullying and a concern with school health culminated in 2007 with the implementation of the *Programa Saúde na Escola *[Health Program at School], an inter-sector policy promoting the delivery of integral healthcare to school-aged children and adolescents. According to this proposal, primary healthcare (PHC) teams must put into practice actions that are focused on the promotion of health according to the principles and guidelines of the Brazilian Unified Health System (SUS), addressing the dimensions of a culture of peace and fighting the various expressions of violence within schools and the community^(^ 13 ^)^.

Therefore, identifying the causes and reasons students become involved with bullying is essential to implementing coping strategies focused on human development and health promotion in the school context. From this perspective, this study's aim was to identify the reasons associated with school bullying reported by adolescents in public and private schools in Brazil.

## Method

### Study's design

This cross-sectional study used data from the National Survey of School Health (PeNSE), conducted in 2012. PeNSE addressed behavioral factors of risk and health protection in a sample of 8^th^ grade students attending daytime programs of public and private schools located in urban or rural areas from the entire Brazilian territory. The 9^th^ grade was chosen because it is the minimum level of education required to complete the self-administered questionnaire during data collection.

### Study setting and sampling

The 2010 School Census was used to select the sample and those schools reporting 9^th^ grade classes administered during daytime hours were included in the list; nighttime programs were excluded. The sample was sized to estimate population parameters (proportions or prevalence) in diverse geographic domains comprising the 26 state capitals along with the Federal District; the set of capitals; the five geographic regions (North, Northeast, Southeast, South, and Midwest) in addition to the country as a whole. A probabilistic sample was used and the sampling plan was formed by schools (primary sampling units) and the schools' classes (secondary sampling units). In the case of non-capital cities, the primary sampling units were groups of cities and the secondary sampling units were schools, while classrooms were the tertiary sampling units. A total of 134,310 9^th^ grade students were enrolled in the selected classes administered during daytime hours by public and private schools located in urban and rural areas in the entire Brazilian territory. Of these, 132,123 students were considered regular students and 110,873 were present in classrooms on the day the questionnaire was applied. The final sample included 109,104 students, i.e., 83% of those considered eligible for the study^(^ 14 ^)^.

A total of 86% of students in the sample surveyed in 2012 were between 13 and 15 years old; 47.8% were male and 52.2% were female; and 17.2% students were from private schools and 82.8% were from public schools^(^ 14 ^)^.

### Procedures

Data were collected using smartphones, which were included in the structured, self-applied questionnaires with thematic modules that varied in the number of questions contained. Bullying was one of the dimensions addressed. Data collection was implemented by previously trained agents from the Brazilian Institute of Geography and Statistics (IBGE), in schools during classes from April to September 2012. Further details concerning the methodology can be obtained in specific publications^(^ 14 ^)^.

### Studied variables

The variable bullying was obtained through the question: "How often did some of your friends belittle, mock, scorn, intimidate or scoff at you IN THE LAST 30 DAYS to the point that you became hurt, bothered, annoyed, offended, or humiliated? The answers were categorized as NO (never, rarely, sometimes) and YES (most of the time, always). 

Reasons/causes related to why one experiences bullying were verified through the question: "What is the reason/cause your friends have belittled, mocked, scorned, intimidated or scoffed at you IN THE LAST 30 DAYS?" The answers to this question were analyzed according to the following options: (a) My race or color; b) My religion; c) The appearance of my face; d) the appearance of my body; e) My sexual orientation; f) My region of origin; g) Other reasons.

### Statistical Analysis

The analysis was performed through the computation of the prevalence of the variables experiencing bullying and their respective confidence intervals of 95%, according to the sociodemographic aspects of experiencing bullying (sex, age, race/color, religion, public or private school, mother's education). The reasons/causes of experiencing bullying reported by the students were analyzed according to sociodemographic aspects stratified by race or color, religion, facial appearance, body appearance, sexual orientation, region of origin, others.

When the reason one experienced bullying was reported to be the appearance of body, it was cross-tabulated with the variable Body Image, which was verified by the question: In regard to your body, do you consider yourself: Too thin, Thin, Normal, Fat, Too fat?

These analyses were performed using SPSS, version 20, with the Complex Samples Module, appropriate for data analyses obtained by a complex sampling plan^(^ 15 ^)^.

### Ethical issues

This study was approved by the Institutional Review Board according to referee report No. 192/2012 Registry No. 16805, CONEP/MS on March 27, 2012.

## Results

Table 1 shows that 7.2% (CI95% 6.6-7.8) of the students reported having experienced bullying, always or almost always felt humiliated, by schoolmates. The percentages were higher among male students, 7.9% (CI95% 7.0-9.1), in comparison to female students, 6.5% (CI95% 6.2-6.7); among students whose mothers were characterized by low levels of education, 8.3% (CI95% 7.2-9.4); among those who reported themselves to be Afro-descendant, 8.1% (CI 95%: 7.2-9.1); and among those self-reported as indigenous people 7.9% (CI95%: 7.3-8.5). No difference was found between private schools, 7.6% (CI95%: 6.9-8.3) and private school students, 7.1% (CI95%: 6.2-8.0).

**Table 1 -:** Prevalence of experience of bullying among 9th grade students according to sex, race/color, type of school, and mothers' education. Brazil, 2012 (N=109,104)

		Experiencing Bullying		
		%	Lower limit	Upper Limit
Total		7.2	6.6	7.8
Age (years)				
	<13 years old	8.8	6.6	11.8
	13 years old	7.9	7.6	8.3
	14 years old	7.1	6.5	7.9
	15 years old	6.7	5.6	7.9
	16 years old or older	6.5	6.1	7
Sex				
	Male	7.9	7	9.1
	Female	6.5	6.2	6.7
Race				
	Caucasian	7.3	6.3	8.4
	Mixed	6.6	6.1	7.1
	Afro-descendant	8.1	7.2	9.1
	Asian	8.3	6.9	9.9
	Indigenous	7.9	7.3	8.5
School				
	Public	7.1	6.2	8
	Private	7.6	6.9	8.3
Mother’s education				
	None	8.3	7.2	9.4
	Incomplete middle school	6.5	5.6	7.5
	Complete middle school	6.9	5.3	9.1
	Incomplete high school	7.2	6.1	8.6
	Complete high school	7.2	6.5	8.1
	Some undergraduate studies	7.3	6.3	8.4
	Bachelor’s degree	7.1	6.5	7.7

Most times, 51.2% (CI95% 48.6-53.7%), causes of bullying were not identified followed by body image or appearance, 18.6% (CI95% 16.5-21); facial appearance, 16.2% (15.4%-17.1%); race or color, 6.8% (CI95% 6.4-7.3); sexual orientation, 2.9% (CI95% 2.5-3.5); religion, 2.5% (CI95% 1.9-3.2); and region of origin, 1.7 (CI95%1.5-2). The frequencies of those reporting having experienced bullying and those reporting always or almost always experienced bullying in the last 30 days were similar, except for those reporting the reason was their race/color, among whom frequency increased to always, as shown in Table 2.

**Table 2 -:** Frequency of the main causes/reasons reported by 9th grade students for having experienced bullying. Brazil, 2012 (N=109.104)

Causes for having experienced bullying	Almost Always				Always in the last 30 days				Experiencing Bullying		
	%	CI* 95%			%	CI* 95%			%	CI* 95%	
		LL^†^	UL^‡^			LL^†^	UL^‡^			LL^†^	UL^‡^
My race or color	4.9	4.3	5.5		8.6	7.9	9.3		6.8	6.4	7.3
My religion	2.7	1.8	3.9		2.3	1.9	2.7		2.5	1.9	3.2
The appearance of my face	16.9	14.7	19.3		15.7	14.2	17.2		16.2	15.4	17.1
The appearance of my body	18.9	13.7	25.6		18.4	16.9	19.9		18.6	16.5	21
My sexual orientation	2.5	1.6	4		3.3	2.5	4.4		2.9	2.5	3.5
My region of origin	1.9	1.5	2.5		1.6	1.3	1.8		1.7	1.5	2
Other causes/reasons	52.2	47.8	56.5		50.2	48.9	51.6		51.2	48.6	53.7

* Confidence Interval

† Lower Limit

‡ Upper Limit

Body appearance was cross-tabulated with the variable body image for those reporting that the appearance of their bodies was the reason they suffered bullying, which showed bullying was more frequent among those reporting they were either too fat or too thin, 19.2% (CI95% 15.1-24) and 12.1% (CI95% 10.4-14.0), respectively (Table 3).

**Table 3 -:** Prevalence of bullying among 9th grade students according to body image. Brazil, 2012

Body image	%	95% Confidence Interval	
		Lower Limit	Upper Limit
Too thin	12.1	10.4	14.0
Thin	7.3	5.8	9.2
Normal	6.0	5.5	6.5
Fat	9.6	9.2	10.0
Too fat	19.2	15.1	24.0
Total	7.2	6.6	7.8

The reasons did not vary according to age, except in regard to sexual orientation among students younger than 13 years of age (15% - CI95%: 7.2-28.6). In regard to sex, boys were more frequently bullied than girls and also more frequently reported experiencing bullying triggered by their race or color 8.9% (CI95% 8.19-9.9), while 4.5% (CI95% 3.8-5.2) of the girls reported bullying was triggered for this reason. A total of 3.9% (CI95% 3.5-4.5) of the boys and 1.8% (CI95% 1.2-2.0) of the girls reporting bullying was triggered by their sexual orientation. Race/color shows considerable difference in regard to how often bullying is experienced: Afro-descendant boys report four times more bullying, 23.2% (CI95% 21.8-24.7), while indigenous students report bullying at twice the frequency, 12.5% (CI95%7.5-20.3). Students of mixed race (3.8% CI95% 2.9-4.8), Caucasian (3.1% CI95% 2.5-3.9), and Asian (4.7% CI95% 1.4-14.4), reported bullying is experienced less frequently. Public schools also present a higher number of reports of bullying triggered by race/color, 7.2% (CI95% 6.6-8.0). Bullying triggered by race/color also increased among children of mothers with no education, 11.6% (CI95% 8.5-15.6), as shown in Table 4.

**Table 4 -:** Main causes triggering bullying according to 9th grade students, according to age, sex, race, type of school, mother's level of education. National Survey of School Health, Brazil, 2012

		My race/color				My religion				The appearance of my face				The appearance of my body				My sexual orientation				My region of origin				Other causes/reasons		
		%	LL*	UP^†^		%	LL*	UP^†^		%	LL*	UP^†^		%	LL*	UP^†^		%	LL*	UP^†^		%	LL*	UP^†^		%	LL*	UP^†^
Total		6.8	6.4	7.3		2.5	1.9	3.2		16.2	15.4	17.1		18.6	16.5	21.0		2.9	2.5	3.5		1.7	1.5	2.0		51.2	48.6	53.7
Age																												
	< 13 years old	4.7	1.1	17.8						18.7	7.1	40.7		21.0	13.1	31.8		15.0	7.2	28.6		0.7	0.1	3.4		40.0	32.1	48.5
	13 years old	4.7	3.3	6.7		2.0	1.3	3.1		17.4	14.6	20.5		20.6	18.5	22.9		3.3	2.2	4.8		1.6	0.8	3.2		50.5	48.1	52.9
	14 years old	6.6	5.5	7.9		2.4	2.0	2.9		15.8	14.6	17.1		19.4	16.5	22.8		2.3	1.7	3.0		1.1	0.5	2.3		52.3	50.1	54.5
	15 years old	9.5	6.2	14.3		1.9	1.4	2.6		15.9	14.0	18.0		15.6	11.8	20.3		3.2	1.9	5.3		1.9	0.5	7.0		52.0	45.0	58.9
	16 years old or older	8.3	6.5	10.6		4.6	2.9	7.3		15.6	12.4	19.5		15.8	13.0	19.0		3.6	2.1	5.9		4.3	2.5	7.4		47.7	39.7	55.9
Sex																												
	Male	8.9	8.1	9.9		2.1	1.6	2.9		18.2	16.1	20.6		17.0	15.7	18.4		3.9	3.5	4.5		1.9	1.7	2.2		47.9	44.2	51.6
	Female	4.5	3.8	5.2		2.8	2.1	3.8		14.0	11.6	16.8		20.5	17.4	24.0		1.8	1.2	2.7		1.5	1.2	2.0		54.8	52.6	57.1
Race																												
	Caucasian	3.1	2.5	3.9		1.7	0.9	3.3		16.2	14.9	17.7		21.1	20.1	22.1		3.0	2.6	3.6		1.7	1.3	2.3		53.1	50.1	56.2
	Afro-descendant	23.2	21.8	24.7		2.3	1.4	3.8		16.0	12.5	20.4		14.7	12.5	17.1		2.0	1.1	3.3		1.1	0.4	3.1		40.7	34.4	47.4
	Asian-descendent	4.7	1.4	14.4		1.6	0.4	6.6		18.4	10.8	29.5		14.5	11.6	18.1		3.0	1.4	6.3		2.9	0.8	9.2		54.9	50.2	59.5
	Mixed	3.8	2.9	4.8		3.4	2.3	4.8		15.9	12.3	20.2		18.9	13.8	25.3		2.9	2.2	3.8		1.9	1.4	2.5		53.3	51.2	55.4
	Indigenous	12.5	7.5	20.3		1.8	0.7	4.5		18.1	14.9	21.8		13.3	10.7	16.5		6.2	1.6	20.7		1.6	1.0	2.8		46.4	41.0	51.8
Type of school																												
	Private	5.1	3.8	6.7		1.2	0.7	2.2		16.5	11.5	23.1		20.7	19.0	22.6		2.5	1.3	4.7		1.8	1.0	3.5		52.1	42.8	61.4
	Public	7.2	6.6	8.0		2.7	2.1	3.6		16.2	14.5	18.0		18.2	15.9	20.6		3.0	2.5	3.6		1.7	1.3	2.3		50.9	49.4	52.4

* LL=Lower Limit

† UL=Upper Limit

## Discussion

This study's findings show that 7.2% of the students experienced bullying, which was more frequently reported by younger boys, whose mothers present lower levels of education, and are of Afro-descent or indigenous. Most did not report the reason or cause that triggers bullying. In regard to differences between sexes, the causes reported by boys and girls were similar, mostly appearance of the face and body, however, boys most frequently reported bullying triggered by race/color and sexual orientation. 

This study highlights that "other reasons/causes" is the most frequent option chosen to explain bullying. The frequency with which this option was chosen may be due to the poor understanding of students concerning the process of victimization or how they qualify jokes or the experience of being bullied. The process of victimization is characterized by receiving negative attention or aggressive behavior from peers over time and what determines its occurrence is being different or behaving differently others^(^ 2 ^)^. Investigating what causes the phenomenon based on self-reports addresses these dimensions and the sensitive nature of the issues implicated in the issue.

Almost a fifth of the students reported body appearance, followed by facial appearance, as being causes of bullying. Similar results were found in other contexts that indicate that physical appearance is one of the main reasons a student becomes a victim of bullying^(^ 16 ^)^. A potential interpretation for this information involves culturally valued social standards in which diversity and differences are not tolerated. One epidemiological study conducted with 1,230 students from a city in Rio Grande do Sul, Brazil, identified that 30.1% were overweight or obese, showing that students dissatisfied with their body image were three times more likely to be victims of bullying. Statistically, however, excess weight was not significantly associated with the phenomenon^(^ 16 ^)^. In turn one study, similar to this study, that was developed in Ireland reports that body image, such as considering oneself to be thin or too thin, was significantly associated with being a victim^(^ 17 ^)^.

Classical studies addressing this phenomenon do not report evidence that body image is a determinant factor in the process of victimization^(^ 1 ^)^. Other studies however, verify that victims often present characteristics that distinguish them from most of their peers, such as obesity, thinness, or the use of prosthetics or orthotics^(^ 18 ^-^ 19 ^)^.

A student's skin color or race was also reported as being significantly associated with victimization. Afro-descendant students were four times more likely to experience bullying, while indigenous people were two times more likely to experience bullying. This dimension is also linked to social and cultural issues, to racism and prejudice, since there is a hegemonic pattern of valuing white skin^(^ 20 ^-^ 23 ^)^. One study in the United States correlated race with gender and identified that these variables were significant predictors of bullying. The study shows that boys were 25.5% more likely to become victims than girls, while Afro-descendant students were 46.3% more likely to become victims at school than Caucasian students^(^ 23 ^)^. Afro-descendant and indigenous students addressed in this study were also more likely to become victims due to their race/color. It is worth noting that individuals of mixed race did not present the same rates of being bullied, an aspect that shows the importance of verifying whether students from different races have different criteria to identify and assess violent practices. 

We cannot ignore the factors and individual variables that explain the phenomenon, as we cannot ignore contextual factors, such as mother's education, in the determination of bullying. As observed, the indication of no maternal education was the most prevalent for victimization and the scientific literature considers this variable to be a demographic predictor of students' success or failure at school. One study recently conducted in the United States reports that students whose mothers presented low levels of education were more likely to become victims^(^ 12 ^)^. In general, results concerning association between mother's education and involvement with bullying are explored because the mother's education is considered to be relevant within the families' set of social and cultural characteristics.

Other issues, such as the students' sexual orientation, religion and region of origin, are not shown to be expressive causes for victimization. In fact, these individual characteristics of students are less frequently observed than other characteristics. Nonetheless, they are manifested differently between sexes; for instance, boys more frequently report victimization associated with sexual orientation than do girls. Additionally, the literature shows that sexual orientation is one of the reasons related to bullying^(^ 11 ^)^. Therefore, these are important variables through which the phenomenon may be approached and related to proposing interventions intended to understand diversity, especially considering the diffuse nature of these in modern times and the emergence of other expressions of sexuality, religiosity, and migratory movements that require understanding and tolerance of diversity^(^ 4 ^,^ 23 ^-^ 24 ^)^.

Overall, the results are relevant and contribute to the understanding of bullying and enable discussing the problem of violence within the school environment. Bullying is manifested through different signs, behaviors, and prejudice in interpersonal relationships among students. Because of its specificity and complexity, bullying in an interdisciplinary and inter-sector object that demands solutions follow the same logic and direction, such as the Health School Project. Education actions and health promotion at school are different ways for PHC workers to encourage new forms in which students may relate with each other and with the world^(^ 10 ^,^ 13 ^)^.

Finally we mention some of the study's limitations. Despite the survey's validity and reliability, its cross-sectional design hinders causal/temporal inferences between exposure to or involvement with school bullying. This limits addressing the issue of causality, though this study's results agree with those reported by prospective studies. In this sense, the individual characteristics of victims do not justify aggressive and violent behavior that is inherent to bullying, as they cannot be isolated, assessed and exclusively seen as causes or motivations to become involved with the phenomenon. Another limitation is the large number of references to the option "other causes/reasons" in the experience of bullying. Hence, we suggest that other psychological characteristics or social relationships be addressed by the instrument, such as shyness, reservation in resolving conflicts, low self-esteem, among other factors. Additionally, students should be asked to indicate causes and reasons they suffer bullying even after providing alternative answers, as an opportunity to fill in some of the gaps observed. 

## Conclusions

This study's results concerning the identification of reasons associated with bullying among Brazilian students show that some individual characteristics are related to the phenomenon and contextual aspects that determine it. Bullying is a common experience in the lives of Brazilian students and a problem within the domain of the health field since it gathers determinants of the health-disease-care process for school-aged children and adolescents. This debate is highly important because it support tools for the development of other studies and health practices, especially in primary healthcare and in the interface between health and education. 

We expect these data to encourage attention being paid to public policies concerning this issue, resulting in indicators being provided that can support the development of coping strategies at the inter-sector and inter-disciplinary levels, with a view to encourage a non-violent culture, partnering the health and education sectors. Further studies are needed, especially those providing qualitative analyses or triangulation methods and approaches, to understand the meanings and processes in which bullying emerges in the school context and its dynamics in the reality of Brazilian schools.
